# Flubenziamide (Pesticides)

**DOI:** 10.14252/foodsafetyfscj.2018011s

**Published:** 2019-03-29

**Authors:** Food Safety Commission of Japan

## Abstract

The Food Safety Commission of Japan (FSCJ) conducted a risk assessment of flubendiamide (CAS No. 272451-65-7), an iodophthalimide insecticide for the setting of an acceptable daily intake (ADI) in 2006. FSCJ now has assessed this insecticide for the setting of an acute reference dose (ARfD). Data including fate in animals (rats and mice) and residues in crops (burdock roots, pumpkins and others) were newly submitted. Major adverse effects of flubendiamide include hepatocellular hypertrophy, fatty changes in hepatocytes, follicular epithelial cell hypertrophy in thyroid and ocular enlarged eye in rats. No neurotoxicity, carcinogenicity, reproductive toxicity, teratogenicity, neurodevelopmental toxicity and genotoxicity were observed. The lowest no-observed-adverse-effect level (NOAEL) in the toxicological studies was 1.70 mg/kg body weight/day in a two-year carcinogenicity study in rats. FSCJ confirmed an ADI of 0.017 mg/kg bw/day after applying a safety factor of 100 to the NOAEL. Adverse effects elicited by a single oral administration of flubendiamide would be abnormalities in eyes such as ocular hypertrophy and iris adhesion in offspring, which were obtained in a two-generation reproductive toxicity study, a one-generation reproductive toxicity study and a neurodevelopmental toxicity study in rats. FSCJ judged that these studies may be applicable to set the ARfD for lactating women in relation to the exposure of flubendiamide to offspring after the birth through breast milk. By taking into account the overall evaluations of the two-generation reproductive toxicity study, one-generation reproductive toxicity study and neurodevelopmental toxicity study in rats, FSCJ judged NOAEL of 15.0 mg/kg bw/day as for an overall NOAEL, and consequently specified an ARfD of 0.15 mg/kg bw/day for lactating women by applying a safety factor of 100 to the NOAEL.

## Conclusion in Brief

The Food Safety Commission of Japan (FSCJ) conducted a risk assessment of flubendiamide (CAS No. 272451-65-7), an iodophthalimide insecticide for the setting of an acceptable daily intake (ADI) in 2006. FSCJ now has assessed this insecticide for the setting of an acute reference dose (ARfD). Data including fate in animals (rats and mice) and residues in crops (burdock roots, pumpkins and others) were newly submitted.

The data used in the assessment include on the fate in animals (rats, mice, goats and chickens), fate in plants (apples, cabbage and others), residues in crops, subacute toxicity (rats, mice and dogs), chronic toxicity (rats and dogs), carcinogenicity (rats and mice), two-generation and one-generation reproductive toxicity (rats), developmental toxicity (rats and rabbits), developmental neurotoxicity (rats) and genotoxicity.

Major adverse effects of flubendiamide include hepatocellular hypertrophy, fatty changes in hepatocytes, follicular epithelial cell hypertrophy in thyroid and ocular enlarged eye in rats. No neurotoxicity, carcinogenicity, reproductive toxicity, teratogenicity, neurodevelopmental toxicity and genotoxicity were observed.

Based on the above results, flubendiamide (parent compound only) was identified as the substance relevant to the residue definition for dietary risk assessment in agricultural products and livestock products.

The lowest no-observed-adverse-effect level (NOAEL) in the toxicological studies was 1.70 mg/kg body weight/day in a two-year carcinogenicity study in rats. FSCJ confirmed an ADI of 0.017 mg/kg bw/day after applying a safety factor of 100 to the NOAEL.

Adverse effects elicited by a single oral administration of flubendiamide would be abnormalities in eyes such as ocular hypertrophy and iris adhesion in offspring, which were obtained in a two-generation reproductive toxicity study, a one-generation reproductive toxicity study and a neurodevelopmental toxicity study in rats. FSCJ judged that these studies may be applicable to set the ARfD for lactating women in relation to the exposure of flubendiamide to offspring after the birth through breast milk. Among these studies, the lowest NOAEL was 3.95 mg/kg bw/day in the two-generation reproductive toxicity study and the lowest LOAEL was 99.5 mg/kg bw/day in the neurodevelopmental toxicity study. Moreover, the NOAEL of 15.0 mg/kg bw/day was obtained in the one-generation reproductive toxicity study. These values assumingly came from the dose settings of studies above. By taking into account the overall evaluations of the two-generation reproductive toxicity study, one-generation reproductive toxicity study and neurodevelopmental toxicity study in rats, FSCJ judged NOAEL of 15.0 mg/kg bw/day as for an overall NOAEL, and consequently specified an ARfD of 0.15 mg/kg bw/day for lactating women by applying a safety factor of 100 to the NOAEL. For general population, FSCJ judged it unnecessary to specify the ARfD, since no adverse effects would be likely to be elicited by a single oral administration of flubendiamide.

**Table 1.** *Levels relevant to toxicological evaluation of flubendiamide*

Species	Study	Dose (mg/kg bw/day)	NOAEL (mg/kg bw/day)	LOAEL (mg/kg bw/day)	Critical endpoints^1^
Rat	90-day subacute toxicity study	0, 20, 50, 200, 2 000, 20 000 ppm	M: 11.4 F: 3.29	M: 116 F: 13.1	M: Increase in PLT F: Centrilobular hepatocellular fatty change and others
		M: 0, 1.15, 2.85, 11.4, 116, 1 190 F: 0, 1.30, 3.29, 13.1, 128, 1 320			
	One-year chronic toxicity study	0, 20, 50, 2 000, 20 000 ppm	M: 1.95 F: 2.40	M: 79.3 F: 97.5	FM: Follicular cell hypertrophy and others
		M: 0, 0.781, 1.95, 79.3, 822 F: 0, 0.960, 2.40, 97.5, 998			
	Two-year carcinogenicity study	0, 50, 1 000, 20 000 ppm	M: 1.70 F: 2.15	M: 33.9 F: 43.7	FM: Centrilobular hepatocellular fatty change and others (Not carcinogenic)
		M: 0, 1.70, 33.9, 705 F: 0, 2.15, 43.7, 912			
	Two-generation reproductive toxicity study	0, 20, 50, 2 000, 20 000 ppm	Parent and Offspring PM: 3.30 PF: 3.95 F_1_M: 4.05 F_1_F: 4.59	Parent and Offspring PM: 131 PF: 159 F_1_M: 162 F_1_F: 176	Parent FM: Follicular cell hypertrophy and others Offspring FM: Increased absolute/relative liver weights and others (No effect on reproduction)
		PM: 0, 1.30, 3.30, 131, 1 310 PF: 0, 1.59, 3.95, 159, 1 580 F_1_M: 0, 1.64, 4.05, 162, 1 640 F_1_F: 0, 1.84, 4.59, 176, 1 810			
	One-generation reproductive toxicity study	0, 50, 200, 2 000, 20 000 ppm	Parent PM: 127 PF: 3.84 F_1_M: 15.9 F_1_F: 5.28 Offspring PM: 12.9 PF: 15.0	Parent PM: 1 290 PF: 15.0 F_1_M: 160 F_1_F: 21.0 Offspring PM: 127 PF: 149	Parent M: Decrease in absolute/relative pituitary weights and others F: Increase in absolute/relative kidney weights and others Offspring FM: Increase in absolute/relative liver weights and others (No effect on reproduction)
		PM: 0, 3.25, 12.9, 127, 1 290 PF: 0, 3.84, 15.0, 149, 1 490 F_1_M: 0, 4.05, 15.9, 160, 1 610 F_1_F: 0, 5.28, 21.0, 206, 2 090			
	Developmental toxicity study	0, 10, 100, 1 000	Maternal: 10 Embryo/fetus: 1 000	Maternal: 100 Embryo/fetus: -	Maternal: Increase in absolute/relative liver weights and others Embryo/fetus: No toxicity (Not teratogenic)
	Neurodevelopmental toxicity study	0, 120, 1 200, 12 000 ppm	Maternal and Offspring: 9.9	Maternal and Offspring: 99.5	Maternal: Centrilobular hypertrophy of hepatocytes and others Offspring: Delayed preputial separation and others (No neurodevelopmental toxicity)
		F: 0, 9.9, 99.5, 980			
Mouse	90-day subacute toxicity study	0, 50, 100, 1 000, 10 000 ppm	M: 11.9 F: 14.7	M: 123 F: 145	FM: Centrilobular hypertrophy of hepatocytes and others
		M: 0, 6.01, 11.9, 123, 1 210 F: 0, 7.13, 14.7, 145, 1 420			
	18-month carcinogenicity study	0, 50, 1 000, 10 000 ppm	M: 4.85 F: 4.44	M: 94 F: 93	FM: Thyroid follicular cell hypertrophy and others (Not carcinogenic)
		M: 0, 4.85, 94, 988 F: 0, 4.44, 93, 937			
Rabbits	Developmental toxicity study	0, 20, 100, 1 000	Maternal: 100 Embryo/fetus: 1 000	Maternal: 1 000 Embryo/fetus: -	Maternal: Decreased feed consumption and others Embryo/fetus: No toxicity (Not teratogenic)
Dog	90-day subacute toxicity study	0, 100, 2 000, 40 000 ppm	M: 2.58 F: 2.82	M: 52.7 F: 59.7	FM: Increase in absolute/relative adrenal weights and others
		M: 0, 2.58, 52.7, 1 080 F: 0, 2.82, 59.7, 1 140			
	One-year chronic toxicity study	0, 100, 1 500, 20 000 ppm	M: 2.21 F: 2.51	M: 35.2 F: 37.9	M: Increase in relative liver weights and others F: Increase in ALP and others
		M: 0, 2.21, 35.2, 484 F: 0, 2.51, 37.9, 533			
ADI			NOAEL: 1.70 SF: 100 ADI: 0.017		
The critical study for setting the ADI			Two-year carcinogenicity study in rats		

M, Male; F, Female; F/M, both sexes; ADI, Acceptable daily intake; NOAEL, No-observed-adverse-effect level; SF, Safety factor;

-: Lowest-observed-adverse-effect level (LOAEL) was not derived

^1^The adverse effect observed at LOAEL

**Table 2.** *Adverse effects possibly elicited by a single oral administration (Lactating women)*

Species	Study	Dose (mg/kg bw/day)	NOAEL and end point for establishing ARfD^1)^ (mg/kg bw/day)
Rat	Two-generation reproductive toxicity study	0, 20, 50, 2 000, 20 000 ppm	PF: 3.95 F_1_F: 4.59 F_1_ and F_2_ Offspring FM: Enlargement of eyeball, adhesion and bleeding of iris, hydropic degeneration of basal layer of the corneal epithelium, corneal epithelial vacuolation, keratitis, iritis and cataract
		PF: 0, 1.59, 3.95, 159, 1 580 F_1_F: 0, 1.84, 4.59, 176, 1 810	
	One-generation reproductive toxicity study	0, 50, 200, 2 000, 20 000 ppm	PF: 15.0 F_1_ Offspring FM: Adhesion and bleeding of iris, iritis and cataract
		PF: 0, 3.84, 15.0, 149, 1 490	
	Neurodevelopmental toxicity study	0, 120, 1 200, 12 000 ppm	Maternal: 9.9 Offspring FM: Enlargement of eyeball, corneal opacity, exophthalmos, and anterior iris adhesion
		Maternal: 0, 9.9, 99.5, 980	
ARfD			NOAEL: 15.0 SF: 100 ARfD: 0.15
The critical study for setting ARfD			Overall evaluation of two-generation reproductive toxicity study, one-generation reproductive toxicity study and neurodevelopmental toxicity study in rats

PF, Female in P generation; F_1_F, Female in F_1_ generation; F_2_, F_2_ generation;

ARfD, Acute reference dose; NOAEL, No-observed-adverse-effect level; SF, Safety factor;

^1)^, The adverse effect observed at LOAEL

